# 2-Bromo­beclometasone dipropionate

**DOI:** 10.1107/S1600536809025987

**Published:** 2009-07-11

**Authors:** Kamal Aziz Ketuly, A. Hamid A. Hadi, Seik Weng Ng

**Affiliations:** a Department of Chemistry, University of Malaya, 50603 Kuala Lumpur, Malaysia

## Abstract

In the crystal structure of 2-bromo­beclometasone dipropionate [systematic name: (8*S*,9*R*,10*S*,11*S*,13*S*,14*S*,16*S*,17*R*)-2-bromo-9α-chloro-11-hydr­oxy-10,13,16-trimethyl-3-oxo-17-[2-(propion­yloxy)acet­yl]-6,7,8,9,10,11,12,13,14,15,16,17-dodeca­hydro-3*H*-cyclo­penta­[*a*]phenanthren-17-yl propionate], C_28_H_36_BrClO_7_, the six-membered ring with the 1,4-diene-3-one composition is planar (r.m.s. deviations = 0.03 and 0.04 Å for the two independent mol­ecules), whereas the remaining six-membered rings have chair conformations. Each of the independent mol­ecules self-associates *via* O—H⋯O_propionate_ hydrogen bonding, generating a supra­molecular chain running along the *b* axis. The crystal is twinned, with the monoclinic unit cell emulating an orthorhomic crystal system; the major twin component constitutes approximately 60%.

## Related literature

For the NMR and crystal structure of the asthma drug beclometasone dipropionate monohydrate, see: Othman *et al.* (2008 ▶); Duax *et al.* (1981 ▶).
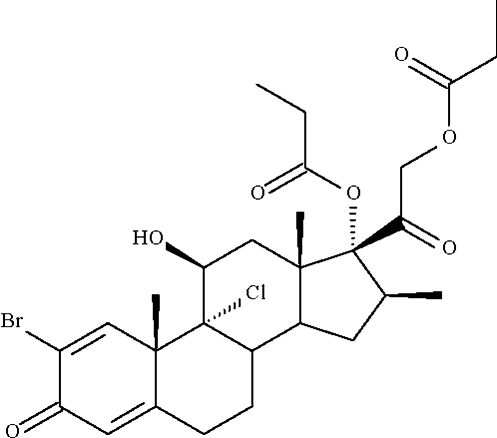


## Experimental

### 

#### Crystal data

C_28_H_36_BrClO_7_
*M* *_r_* = 599.93
Monoclinic, 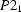

*a* = 12.0999 (3) Å
*b* = 13.8673 (3) Å
*c* = 16.7971 (4) Å
β = 90.106 (1)°
*V* = 2818.4 (1) Å^3^
*Z* = 4
Mo *K*α radiation
μ = 1.60 mm^−1^
*T* = 140 K
0.22 × 0.11 × 0.02 mm

#### Data collection

Bruker SMART APEX diffractometer
Absorption correction: multi-scan (*SADABS*; Sheldrick, 1996 ▶) *T*_min_ = 0.642, *T*_max_ = 0.746 (expected range = 0.834–0.969)
23151 measured reflections
12754 independent reflections
9841 reflections with *I* > 2σ(*I*)
*R*_int_ = 0.045

#### Refinement

*R*[*F* ^2^ > 2σ(*F* ^2^)] = 0.046
*wR*(*F* ^2^) = 0.086
*S* = 0.94
12754 reflections
680 parameters
1 restraint
H-atom parameters constrained
Δρ_max_ = 0.61 e Å^−3^
Δρ_min_ = −0.52 e Å^−3^
Absolute structure: Flack (1983 ▶), 6028 Friedel pairs
Flack parameter: 0.001 (7)

### 

Data collection: *APEX2* (Bruker, 2008 ▶); cell refinement: *SAINT* (Bruker, 2008 ▶); data reduction: *SAINT*; program(s) used to solve structure: *SHELXS97* (Sheldrick, 2008 ▶); program(s) used to refine structure: *SHELXL97* (Sheldrick, 2008 ▶); molecular graphics: *X-SEED* (Barbour, 2001 ▶); software used to prepare material for publication: *publCIF* (Westrip, 2009 ▶).

## Supplementary Material

Crystal structure: contains datablocks global, I. DOI: 10.1107/S1600536809025987/tk2487sup1.cif

Structure factors: contains datablocks I. DOI: 10.1107/S1600536809025987/tk2487Isup2.hkl

Additional supplementary materials:  crystallographic information; 3D view; checkCIF report

## Figures and Tables

**Table 1:** Hydrogen-bond geometry (Å, °)

*D*—H⋯*A*	*D*—H	H⋯*A*	*D*⋯*A*	*D*—H⋯*A*
O2—H2⋯O7^i^	0.82	2.11	2.817 (5)	145
O9—H9⋯O14^ii^	0.82	1.98	2.754 (5)	158

Symmetry codes: (i) 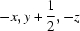; (ii) 
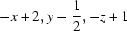
.

**Fig. 1.**
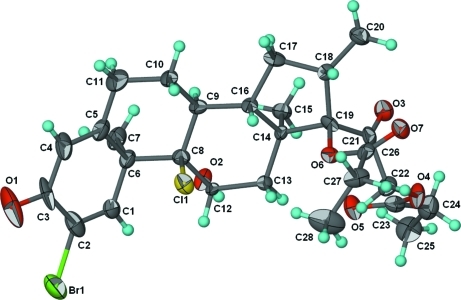
Thermal ellipsoid plot (Barbour, 2001) of one independent molecule of C28H36BrClO7 at the 70% probability level. Hydrogen atoms are drawn as spheres of arbitrary radius.

**Fig. 2.**
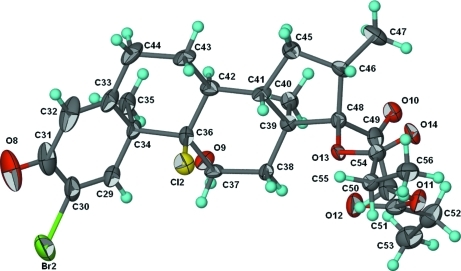
Thermal ellipsoid plot (Barbour, 2001) of the second independent molecule of C28H36BrClO7 at the 70% probability level. Hydrogen atoms are drawn as spheres of arbitrary radius.

## supplementary crystallographic information

### Experimental

The synthesis of the compound is described in a 1988 study commissioned by the Glaxo company (now called Glaxo Smith Kline), External Report No. WAP/88/007.

The brominated compound was isolated from the mother liquor of the first crystallization of beclomethasone dipropionate (10 g). This was dissolved in hexane:ethyl acetate (8:1 v/v) and eluted by flash-column silica gel chromatography with nitrogen at 20 psi. This procedure was repeated for other batches to accumulate more material. The combined fractions were further fractioniated on fresh silica gel. One of the fractions (162 mg) yielded crystals that were recrystallized from hexane:ethyl acetate (3:1 v/v) (47 mg); 490–491 K. Elemental analysis: Calc. for C_28_H_36_BrClO_7_: C 56.19, H 6.02, Br 13.21, Cl, 5.85%. Found: C 56.03, H 6.12, Br 12.97, Cl, 5.76% . MS^+. ^599.

### Refinement

Hydrogen atoms were placed at calculated positions (C–H 0.95–1.00 Å, O–H 0.82 Å) and were treated as riding on their parent carbon atoms, with *U*(H) set to 1.2–1.5 times *U*_eq_(*C*,*O*).

The monoclinic unit cell emulates an orthorhombic unit cell as the β-angle is nearly a right angle. The twin law (-1 0 0 0 -1 0 0 0 1) showed that the major twin component is 60%.

### Crystal data

**Table:**

C_28_H_36_BrClO_7_	*F*(000) = 1248
*M**_r_* = 599.93	*D*_x_ = 1.414 Mg m^−^^3^
Monoclinic, *P*2_1_	Mo *K*α radiation, λ = 0.71073 Å
Hall symbol: P 2yb	Cell parameters from 4369 reflections
*a* = 12.0999 (3) Å	θ = 2.5–22.4°
*b* = 13.8673 (3) Å	µ = 1.60 mm^−^^1^
*c* = 16.7971 (4) Å	*T* = 140 K
β = 90.106 (1)°	Plate, colorless
*V* = 2818.4 (1) Å^3^	0.22 × 0.11 × 0.02 mm
*Z* = 4	

### Data collection

**Table:**

Bruker SMART APEX diffractometer	12754 independent reflections
Radiation source: fine-focus sealed tube	9841 reflections with *I* > 2σ(*I*)
graphite	*R*_int_ = 0.045
ω scans	θ_max_ = 27.5°, θ_min_ = 1.2°
Absorption correction: multi-scan (*SADABS*; Sheldrick, 1996)	*h* = −15→15
*T*_min_ = 0.642, *T*_max_ = 0.746	*k* = −17→17
23151 measured reflections	*l* = −21→21

### Refinement

**Table:**

Refinement on *F*^2^	Secondary atom site location: difference Fourier map
Least-squares matrix: full	Hydrogen site location: inferred from neighbouring sites
*R*[*F*^2^ > 2σ(*F*^2^)] = 0.046	H-atom parameters constrained
*wR*(*F*^2^) = 0.086	*w* = 1/[σ^2^(*F*_o_^2^) + (0.0055*P*)^2^] where *P* = (*F*_o_^2^ + 2*F*_c_^2^)/3
*S* = 0.94	(Δ/σ)_max_ = 0.001
12754 reflections	Δρ_max_ = 0.61 e Å^−^^3^
680 parameters	Δρ_min_ = −0.52 e Å^−^^3^
1 restraint	Absolute structure: Flack (1983), 6028 Friedel pairs
Primary atom site location: structure-invariant direct methods	Flack parameter: 0.001 (7)

### Fractional atomic coordinates and isotropic or equivalent isotropic displacement parameters (Å^2^)

**Table:**

	*x*	*y*	*z*	*U*_iso_*/*U*_eq_	
Br1	0.54075 (6)	0.49998 (4)	0.19693 (4)	0.04545 (18)	
Br2	0.47284 (6)	0.38183 (4)	0.69368 (4)	0.04703 (18)	
Cl1	0.31003 (12)	0.21093 (9)	0.14232 (8)	0.0266 (3)	
Cl2	0.67988 (12)	0.69926 (9)	0.65968 (7)	0.0273 (3)	
O1	0.6876 (4)	0.3240 (3)	0.2194 (3)	0.0657 (16)	
O2	0.2810 (3)	0.3459 (2)	−0.0630 (2)	0.0241 (9)	
H2	0.2347	0.3857	−0.0769	0.036*	
O3	−0.0895 (3)	0.1241 (3)	−0.1469 (2)	0.0288 (9)	
O4	−0.2209 (3)	0.2617 (3)	−0.0927 (2)	0.0288 (9)	
O5	−0.0790 (3)	0.3584 (3)	−0.1263 (2)	0.0286 (9)	
O6	−0.0029 (3)	0.1104 (2)	0.05595 (19)	0.0188 (8)	
O7	−0.1480 (3)	0.0108 (2)	0.0430 (2)	0.0258 (8)	
O8	0.3216 (5)	0.5484 (4)	0.7329 (3)	0.0651 (15)	
O9	0.7133 (3)	0.5636 (2)	0.4572 (2)	0.0234 (9)	
H9	0.7495	0.5143	0.4507	0.035*	
O10	1.0599 (3)	0.8016 (2)	0.3561 (2)	0.0280 (9)	
O11	1.2091 (3)	0.6755 (3)	0.4091 (2)	0.0289 (9)	
O12	1.0782 (3)	0.5674 (3)	0.3757 (3)	0.0324 (10)	
O13	0.9919 (3)	0.8182 (2)	0.56103 (18)	0.0181 (8)	
O14	1.1285 (3)	0.9212 (2)	0.5346 (2)	0.0237 (8)	
C1	0.4654 (4)	0.3656 (4)	0.0826 (3)	0.0243 (12)	
H1	0.4146	0.4154	0.0693	0.029*	
C2	0.5368 (5)	0.3808 (4)	0.1419 (3)	0.0327 (14)	
C3	0.6175 (5)	0.3074 (4)	0.1685 (4)	0.0369 (16)	
C4	0.6101 (5)	0.2152 (4)	0.1282 (4)	0.0350 (14)	
H4	0.6584	0.1649	0.1446	0.042*	
C5	0.5382 (5)	0.1970 (4)	0.0688 (3)	0.0288 (13)	
C6	0.4617 (4)	0.2731 (3)	0.0360 (3)	0.0223 (12)	
C7	0.5041 (5)	0.2971 (4)	−0.0489 (3)	0.0300 (14)	
H7A	0.5848	0.3024	−0.0480	0.045*	
H7B	0.4721	0.3584	−0.0666	0.045*	
H7C	0.4821	0.2457	−0.0857	0.045*	
C8	0.3392 (4)	0.2315 (3)	0.0368 (3)	0.0195 (11)	
C9	0.3330 (4)	0.1346 (3)	−0.0066 (3)	0.0200 (12)	
H9a	0.3533	0.1462	−0.0635	0.024*	
C10	0.4151 (4)	0.0613 (3)	0.0274 (4)	0.0253 (13)	
H10A	0.3925	0.0441	0.0822	0.030*	
H10B	0.4128	0.0020	−0.0053	0.030*	
C11	0.5332 (5)	0.1001 (4)	0.0290 (4)	0.0338 (14)	
H11A	0.5813	0.0542	0.0581	0.041*	
H11B	0.5612	0.1057	−0.0261	0.041*	
C12	0.2493 (4)	0.3049 (3)	0.0116 (3)	0.0179 (11)	
H12	0.2470	0.3577	0.0521	0.021*	
C13	0.1328 (4)	0.2587 (3)	0.0063 (3)	0.0184 (11)	
H13A	0.1060	0.2460	0.0609	0.022*	
H13B	0.0817	0.3056	−0.0186	0.022*	
C14	0.1283 (4)	0.1650 (3)	−0.0409 (3)	0.0159 (11)	
C15	0.1439 (4)	0.1828 (4)	−0.1301 (3)	0.0227 (12)	
H15A	0.2217	0.1975	−0.1409	0.034*	
H15B	0.0979	0.2374	−0.1468	0.034*	
H15C	0.1222	0.1250	−0.1599	0.034*	
C16	0.2154 (4)	0.0949 (3)	−0.0053 (3)	0.0168 (11)	
H16	0.1955	0.0858	0.0519	0.020*	
C17	0.1884 (4)	0.0007 (4)	−0.0474 (3)	0.0224 (11)	
H17A	0.2214	−0.0548	−0.0189	0.027*	
H17B	0.2160	0.0013	−0.1028	0.027*	
C18	0.0620 (4)	−0.0044 (4)	−0.0457 (3)	0.0165 (10)	
H18	0.0425	−0.0419	0.0031	0.020*	
C19	0.0216 (4)	0.1023 (3)	−0.0301 (3)	0.0180 (11)	
C20	0.0102 (5)	−0.0589 (3)	−0.1159 (3)	0.0266 (13)	
H20A	0.0446	−0.1226	−0.1206	0.040*	
H20B	0.0222	−0.0224	−0.1651	0.040*	
H20C	−0.0693	−0.0664	−0.1068	0.040*	
C21	−0.0750 (4)	0.1418 (3)	−0.0776 (3)	0.0213 (12)	
C22	−0.1497 (4)	0.2152 (4)	−0.0356 (3)	0.0247 (12)	
H22A	−0.1949	0.1820	0.0051	0.030*	
H22B	−0.1036	0.2642	−0.0083	0.030*	
C23	−0.1728 (5)	0.3310 (3)	−0.1386 (3)	0.0238 (12)	
C24	−0.2494 (5)	0.3645 (4)	−0.2015 (4)	0.0364 (14)	
H24A	−0.3242	0.3715	−0.1784	0.044*	
H24B	−0.2535	0.3147	−0.2436	0.044*	
C25	−0.2165 (6)	0.4583 (4)	−0.2385 (4)	0.0375 (15)	
H25A	−0.2678	0.4741	−0.2818	0.056*	
H25B	−0.1414	0.4530	−0.2598	0.056*	
H25C	−0.2187	0.5094	−0.1982	0.056*	
C26	−0.0847 (4)	0.0556 (3)	0.0855 (3)	0.0206 (12)	
C27	−0.0835 (5)	0.0551 (4)	0.1745 (3)	0.0284 (13)	
H27A	−0.0349	0.0021	0.1928	0.034*	
H27B	−0.1591	0.0411	0.1937	0.034*	
C28	−0.0439 (6)	0.1494 (4)	0.2124 (3)	0.0505 (17)	
H28A	−0.0488	0.1444	0.2704	0.076*	
H28B	−0.0905	0.2027	0.1939	0.076*	
H28C	0.0330	0.1615	0.1970	0.076*	
C29	0.5366 (5)	0.5327 (4)	0.5906 (3)	0.0254 (13)	
H29	0.5906	0.4874	0.5737	0.030*	
C30	0.4678 (5)	0.5071 (5)	0.6483 (3)	0.0330 (14)	
C31	0.3842 (6)	0.5721 (5)	0.6799 (4)	0.0439 (18)	
C32	0.3840 (5)	0.6696 (5)	0.6466 (4)	0.0449 (18)	
H32	0.3330	0.7152	0.6672	0.054*	
C33	0.4528 (5)	0.6975 (4)	0.5883 (3)	0.0287 (13)	
C34	0.5335 (4)	0.6289 (3)	0.5506 (3)	0.0212 (12)	
C35	0.4887 (5)	0.6095 (4)	0.4648 (3)	0.0274 (13)	
H35A	0.4128	0.5850	0.4680	0.041*	
H35B	0.5356	0.5616	0.4384	0.041*	
H35C	0.4896	0.6696	0.4342	0.041*	
C36	0.6517 (4)	0.6758 (4)	0.5533 (3)	0.0198 (11)	
C37	0.7467 (4)	0.6092 (3)	0.5292 (3)	0.0198 (12)	
H37	0.7543	0.5583	0.5710	0.024*	
C38	0.8584 (4)	0.6604 (3)	0.5215 (3)	0.0192 (11)	
H38A	0.8863	0.6762	0.5754	0.023*	
H38B	0.9119	0.6158	0.4965	0.023*	
C39	0.8529 (4)	0.7532 (3)	0.4722 (3)	0.0168 (11)	
C40	0.8294 (5)	0.7317 (4)	0.3834 (3)	0.0240 (12)	
H40A	0.7532	0.7088	0.3774	0.036*	
H40B	0.8806	0.6819	0.3645	0.036*	
H40C	0.8396	0.7906	0.3521	0.036*	
C41	0.7662 (4)	0.8197 (3)	0.5097 (3)	0.0190 (11)	
H41	0.7884	0.8306	0.5664	0.023*	
C42	0.6501 (4)	0.7747 (3)	0.5103 (3)	0.0223 (12)	
H42	0.6282	0.7629	0.4537	0.027*	
C43	0.5653 (5)	0.8430 (4)	0.5463 (4)	0.0277 (13)	
H43A	0.5906	0.8627	0.5999	0.033*	
H43B	0.5606	0.9017	0.5129	0.033*	
C44	0.4498 (5)	0.7981 (4)	0.5531 (4)	0.0349 (15)	
H44A	0.4030	0.8399	0.5869	0.042*	
H44B	0.4156	0.7953	0.4995	0.042*	
C45	0.7821 (4)	0.9145 (3)	0.4656 (3)	0.0238 (12)	
H45A	0.7499	0.9690	0.4958	0.029*	
H45B	0.7477	0.9121	0.4121	0.029*	
C46	0.9079 (4)	0.9239 (3)	0.4597 (3)	0.0218 (12)	
H46	0.9313	0.9660	0.5050	0.026*	
C47	0.9425 (5)	0.9759 (4)	0.3841 (3)	0.0341 (14)	
H47A	0.9090	1.0402	0.3830	0.051*	
H47B	0.9177	0.9390	0.3376	0.051*	
H47C	1.0232	0.9820	0.3830	0.051*	
C48	0.9565 (4)	0.8212 (3)	0.4772 (3)	0.0182 (11)	
C49	1.0518 (5)	0.7834 (3)	0.4268 (3)	0.0249 (12)	
C50	1.1341 (5)	0.7155 (4)	0.4666 (3)	0.0272 (12)	
H50A	1.1764	0.7512	0.5076	0.033*	
H50B	1.0936	0.6627	0.4934	0.033*	
C51	1.1673 (5)	0.6009 (4)	0.3650 (3)	0.0229 (12)	
C52	1.2503 (5)	0.5698 (4)	0.3026 (4)	0.0332 (14)	
H52A	1.3252	0.5704	0.3264	0.040*	
H52B	1.2495	0.6168	0.2583	0.040*	
C53	1.2268 (5)	0.4703 (4)	0.2700 (4)	0.0389 (16)	
H53A	1.2865	0.4511	0.2341	0.058*	
H53B	1.1566	0.4712	0.2407	0.058*	
H53C	1.2218	0.4242	0.3141	0.058*	
C54	1.0753 (4)	0.8780 (4)	0.5826 (3)	0.0223 (11)	
C55	1.0950 (4)	0.8777 (4)	0.6712 (3)	0.0205 (11)	
H55A	1.1345	0.8179	0.6863	0.025*	
H55B	1.0230	0.8781	0.6991	0.025*	
C56	1.1626 (4)	0.9647 (3)	0.6976 (3)	0.0291 (12)	
H56A	1.1751	0.9615	0.7552	0.044*	
H56B	1.1224	1.0240	0.6847	0.044*	
H56C	1.2339	0.9646	0.6700	0.044*	

### Atomic displacement parameters (Å^2^)

**Table:**

	*U*^11^	*U*^22^	*U*^33^	*U*^12^	*U*^13^	*U*^23^
Br1	0.0424 (4)	0.0454 (3)	0.0485 (4)	−0.0172 (3)	−0.0005 (3)	−0.0155 (4)
Br2	0.0408 (4)	0.0540 (4)	0.0462 (4)	−0.0229 (3)	−0.0093 (3)	0.0218 (4)
Cl1	0.0282 (7)	0.0265 (7)	0.0252 (7)	−0.0061 (6)	−0.0008 (5)	0.0004 (6)
Cl2	0.0296 (7)	0.0288 (7)	0.0235 (6)	−0.0042 (6)	−0.0012 (6)	−0.0027 (6)
O1	0.061 (3)	0.051 (3)	0.085 (4)	−0.027 (3)	−0.052 (3)	0.023 (3)
O2	0.018 (2)	0.0180 (18)	0.036 (2)	0.0011 (15)	0.0028 (16)	0.0126 (16)
O3	0.027 (2)	0.033 (2)	0.027 (2)	0.0012 (17)	−0.0083 (17)	−0.0044 (18)
O4	0.018 (2)	0.0268 (19)	0.042 (2)	0.0001 (16)	−0.0097 (17)	0.0128 (18)
O5	0.027 (2)	0.029 (2)	0.030 (2)	−0.0105 (16)	−0.0026 (17)	0.0069 (17)
O6	0.023 (2)	0.0160 (16)	0.0180 (17)	−0.0030 (14)	0.0040 (14)	−0.0002 (14)
O7	0.024 (2)	0.0196 (18)	0.033 (2)	−0.0063 (16)	−0.0001 (16)	0.0000 (17)
O8	0.066 (4)	0.075 (3)	0.054 (3)	−0.031 (3)	0.035 (3)	−0.007 (3)
O9	0.020 (2)	0.0162 (18)	0.034 (2)	0.0022 (15)	−0.0036 (17)	−0.0077 (17)
O10	0.027 (2)	0.0278 (19)	0.029 (2)	−0.0021 (16)	0.0040 (17)	0.0047 (17)
O11	0.016 (2)	0.0262 (19)	0.044 (2)	−0.0025 (16)	0.0029 (17)	−0.0063 (18)
O12	0.022 (2)	0.030 (2)	0.045 (3)	−0.0079 (17)	0.0014 (19)	−0.0019 (19)
O13	0.0177 (19)	0.0170 (17)	0.0195 (17)	−0.0057 (14)	−0.0020 (14)	0.0042 (14)
O14	0.027 (2)	0.0197 (17)	0.024 (2)	−0.0121 (16)	0.0016 (17)	0.0034 (16)
C1	0.021 (3)	0.021 (3)	0.031 (3)	−0.006 (3)	0.004 (2)	−0.001 (2)
C2	0.036 (3)	0.030 (3)	0.032 (3)	−0.019 (3)	−0.002 (3)	0.004 (3)
C3	0.031 (3)	0.036 (3)	0.044 (4)	−0.026 (3)	−0.020 (3)	0.019 (3)
C4	0.023 (3)	0.029 (3)	0.053 (4)	−0.010 (3)	−0.006 (3)	0.011 (3)
C5	0.018 (3)	0.027 (3)	0.042 (3)	0.003 (3)	0.001 (3)	0.004 (3)
C6	0.016 (3)	0.021 (3)	0.029 (3)	0.003 (2)	0.000 (2)	0.002 (2)
C7	0.021 (3)	0.033 (3)	0.036 (3)	−0.001 (2)	0.008 (3)	0.005 (3)
C8	0.021 (3)	0.016 (2)	0.021 (3)	0.001 (2)	0.000 (2)	−0.001 (2)
C9	0.021 (3)	0.013 (2)	0.026 (3)	0.003 (2)	0.001 (2)	−0.002 (2)
C10	0.018 (3)	0.013 (2)	0.045 (3)	0.003 (2)	0.000 (3)	−0.001 (3)
C11	0.029 (3)	0.025 (3)	0.047 (4)	0.010 (3)	−0.004 (3)	0.005 (3)
C12	0.011 (3)	0.011 (2)	0.032 (3)	0.0002 (19)	0.003 (2)	−0.005 (2)
C13	0.016 (3)	0.013 (2)	0.026 (3)	0.001 (2)	−0.002 (2)	−0.002 (2)
C14	0.011 (2)	0.013 (2)	0.024 (3)	0.0014 (19)	−0.001 (2)	0.000 (2)
C15	0.025 (3)	0.020 (3)	0.023 (3)	−0.002 (2)	0.004 (2)	−0.003 (2)
C16	0.017 (3)	0.008 (2)	0.026 (3)	0.0011 (19)	0.001 (2)	0.004 (2)
C17	0.033 (3)	0.010 (2)	0.025 (3)	0.000 (2)	0.001 (2)	0.000 (2)
C18	0.018 (3)	0.017 (2)	0.014 (2)	0.000 (2)	0.0024 (19)	0.003 (2)
C19	0.025 (3)	0.017 (2)	0.012 (2)	−0.004 (2)	−0.004 (2)	−0.0023 (19)
C20	0.030 (3)	0.021 (3)	0.029 (3)	−0.004 (2)	0.003 (2)	−0.004 (2)
C21	0.016 (3)	0.020 (3)	0.028 (3)	−0.007 (2)	−0.001 (2)	0.000 (2)
C22	0.022 (3)	0.020 (3)	0.033 (3)	0.003 (2)	0.000 (2)	0.005 (2)
C23	0.028 (3)	0.017 (2)	0.026 (3)	0.001 (2)	0.001 (2)	−0.002 (2)
C24	0.029 (3)	0.040 (4)	0.041 (3)	0.003 (3)	−0.005 (3)	0.001 (3)
C25	0.043 (4)	0.032 (3)	0.037 (3)	0.003 (3)	−0.001 (3)	0.012 (3)
C26	0.017 (3)	0.014 (3)	0.031 (3)	0.005 (2)	0.001 (2)	0.005 (2)
C27	0.038 (3)	0.023 (3)	0.025 (3)	0.004 (2)	0.011 (2)	0.006 (2)
C28	0.087 (5)	0.039 (3)	0.026 (3)	−0.005 (4)	0.012 (3)	0.002 (3)
C29	0.017 (3)	0.028 (3)	0.032 (3)	−0.002 (2)	0.001 (3)	−0.004 (2)
C30	0.021 (3)	0.048 (3)	0.029 (3)	−0.015 (3)	−0.004 (3)	0.008 (3)
C31	0.037 (4)	0.048 (4)	0.046 (4)	−0.024 (3)	0.006 (3)	−0.009 (3)
C32	0.025 (3)	0.052 (4)	0.058 (4)	−0.002 (3)	0.016 (3)	−0.034 (4)
C33	0.015 (3)	0.026 (3)	0.045 (3)	−0.002 (2)	−0.005 (3)	−0.011 (3)
C34	0.012 (3)	0.021 (2)	0.031 (3)	−0.004 (2)	−0.003 (2)	−0.003 (2)
C35	0.018 (3)	0.032 (3)	0.032 (3)	−0.006 (2)	−0.003 (2)	−0.001 (3)
C36	0.016 (3)	0.020 (2)	0.023 (3)	−0.003 (2)	0.003 (2)	−0.003 (2)
C37	0.019 (3)	0.012 (2)	0.028 (3)	0.003 (2)	−0.006 (2)	−0.001 (2)
C38	0.013 (3)	0.016 (2)	0.029 (3)	−0.001 (2)	−0.001 (2)	0.000 (2)
C39	0.018 (3)	0.018 (2)	0.015 (2)	0.000 (2)	−0.001 (2)	−0.002 (2)
C40	0.022 (3)	0.017 (3)	0.033 (3)	−0.004 (2)	−0.004 (2)	0.000 (2)
C41	0.019 (3)	0.010 (2)	0.028 (3)	0.004 (2)	−0.004 (2)	−0.006 (2)
C42	0.023 (3)	0.015 (2)	0.029 (3)	0.002 (2)	−0.004 (2)	0.001 (2)
C43	0.026 (3)	0.018 (2)	0.039 (3)	0.012 (2)	−0.001 (2)	−0.002 (2)
C44	0.017 (3)	0.029 (3)	0.059 (4)	0.009 (2)	−0.003 (3)	−0.010 (3)
C45	0.022 (3)	0.018 (2)	0.032 (3)	−0.002 (2)	−0.003 (2)	0.001 (2)
C46	0.029 (3)	0.015 (2)	0.022 (3)	−0.003 (2)	−0.002 (2)	0.004 (2)
C47	0.045 (4)	0.025 (3)	0.033 (3)	−0.004 (3)	0.001 (3)	0.012 (3)
C48	0.017 (3)	0.016 (2)	0.021 (3)	−0.002 (2)	0.000 (2)	−0.001 (2)
C49	0.022 (3)	0.020 (3)	0.033 (3)	−0.009 (2)	−0.003 (2)	0.000 (2)
C50	0.026 (3)	0.027 (3)	0.029 (3)	−0.002 (3)	0.005 (2)	−0.005 (3)
C51	0.024 (3)	0.016 (2)	0.028 (3)	0.003 (2)	−0.005 (2)	−0.001 (2)
C52	0.026 (3)	0.045 (3)	0.029 (3)	0.001 (3)	−0.005 (3)	−0.005 (3)
C53	0.042 (4)	0.043 (4)	0.033 (3)	0.007 (3)	−0.003 (3)	−0.001 (3)
C54	0.019 (3)	0.015 (2)	0.032 (3)	−0.002 (2)	−0.004 (2)	0.001 (3)
C55	0.019 (2)	0.020 (2)	0.022 (3)	0.001 (2)	−0.0003 (19)	0.000 (2)
C56	0.030 (3)	0.028 (3)	0.029 (3)	0.003 (2)	−0.003 (3)	0.004 (3)

### Geometric parameters (Å, °)

**Table:**

Br1—C2	1.895 (6)	C24—H24A	0.9900
Br2—C30	1.898 (6)	C24—H24B	0.9900
Cl1—C8	1.830 (5)	C25—H25A	0.9800
Cl2—C36	1.847 (5)	C25—H25B	0.9800
O1—C3	1.224 (7)	C25—H25C	0.9800
O2—C12	1.428 (6)	C26—C27	1.495 (7)
O2—H2	0.8200	C27—C28	1.531 (7)
O3—C21	1.202 (6)	C27—H27A	0.9900
O4—C23	1.364 (6)	C27—H27B	0.9900
O4—C22	1.442 (6)	C28—H28A	0.9800
O5—C23	1.215 (6)	C28—H28B	0.9800
O6—C26	1.343 (6)	C28—H28C	0.9800
O6—C19	1.481 (6)	C29—C30	1.328 (8)
O7—C26	1.217 (6)	C29—C34	1.494 (7)
O8—C31	1.214 (8)	C29—H29	0.9500
O9—C37	1.423 (6)	C30—C31	1.455 (9)
O9—H9	0.8200	C31—C32	1.464 (10)
O10—C49	1.217 (6)	C32—C33	1.344 (8)
O11—C51	1.369 (6)	C32—H32	0.9500
O11—C50	1.438 (6)	C33—C34	1.505 (7)
O12—C51	1.187 (6)	C33—C44	1.515 (8)
O13—C54	1.355 (6)	C34—C35	1.562 (7)
O13—C48	1.472 (6)	C34—C36	1.571 (7)
O14—C54	1.194 (6)	C35—H35A	0.9800
C1—C2	1.334 (8)	C35—H35B	0.9800
C1—C6	1.504 (7)	C35—H35C	0.9800
C1—H1	0.9500	C36—C37	1.529 (7)
C2—C3	1.479 (9)	C36—C42	1.551 (7)
C3—C4	1.450 (9)	C37—C38	1.532 (7)
C4—C5	1.346 (8)	C37—H37	1.0000
C4—H4	0.9500	C38—C39	1.531 (7)
C5—C11	1.503 (8)	C38—H38A	0.9900
C5—C6	1.507 (7)	C38—H38B	0.9900
C6—C7	1.552 (8)	C39—C40	1.547 (7)
C6—C8	1.590 (7)	C39—C41	1.533 (7)
C7—H7A	0.9800	C39—C48	1.571 (7)
C7—H7B	0.9800	C40—H40A	0.9800
C7—H7C	0.9800	C40—H40B	0.9800
C8—C9	1.530 (7)	C40—H40C	0.9800
C8—C12	1.549 (7)	C41—C45	1.521 (7)
C9—C16	1.526 (7)	C41—C42	1.536 (7)
C9—C10	1.531 (7)	C41—H41	1.0000
C9—H9a	1.0000	C42—C43	1.523 (7)
C10—C11	1.527 (8)	C42—H42	1.0000
C10—H10A	0.9900	C43—C44	1.534 (8)
C10—H10B	0.9900	C43—H43A	0.9900
C11—H11A	0.9900	C43—H43B	0.9900
C11—H11B	0.9900	C44—H44A	0.9900
C12—C13	1.551 (7)	C44—H44B	0.9900
C12—H12	1.0000	C45—C46	1.531 (7)
C13—C14	1.523 (7)	C45—H45A	0.9900
C13—H13A	0.9900	C45—H45B	0.9900
C13—H13B	0.9900	C46—C47	1.519 (7)
C14—C15	1.531 (7)	C46—C48	1.568 (6)
C14—C16	1.552 (6)	C46—H46	1.0000
C14—C19	1.568 (7)	C47—H47A	0.9800
C15—H15A	0.9800	C47—H47B	0.9800
C15—H15B	0.9800	C47—H47C	0.9800
C15—H15C	0.9800	C48—C49	1.525 (7)
C16—C17	1.521 (7)	C49—C50	1.523 (7)
C16—H16	1.0000	C50—H50A	0.9900
C17—C18	1.531 (7)	C50—H50B	0.9900
C17—H17A	0.9900	C51—C52	1.515 (8)
C17—H17B	0.9900	C52—C53	1.510 (8)
C18—C20	1.534 (7)	C52—H52A	0.9900
C18—C19	1.579 (7)	C52—H52B	0.9900
C18—H18	1.0000	C53—H53A	0.9800
C19—C21	1.517 (7)	C53—H53B	0.9800
C20—H20A	0.9800	C53—H53C	0.9800
C20—H20B	0.9800	C54—C55	1.507 (7)
C20—H20C	0.9800	C55—C56	1.523 (7)
C21—C22	1.534 (7)	C55—H55A	0.9900
C22—H22A	0.9900	C55—H55B	0.9900
C22—H22B	0.9900	C56—H56A	0.9800
C23—C24	1.479 (7)	C56—H56B	0.9800
C24—C25	1.496 (8)	C56—H56C	0.9800
			
C12—O2—H2	109.5	C27—C28—H28C	109.5
C23—O4—C22	115.8 (4)	H28A—C28—H28C	109.5
C26—O6—C19	117.8 (4)	H28B—C28—H28C	109.5
C37—O9—H9	109.5	C30—C29—C34	123.5 (5)
C51—O11—C50	114.9 (4)	C30—C29—H29	118.3
C54—O13—C48	117.0 (4)	C34—C29—H29	118.3
C2—C1—C6	122.8 (5)	C29—C30—C31	122.6 (6)
C2—C1—H1	118.6	C29—C30—Br2	121.2 (5)
C6—C1—H1	118.6	C31—C30—Br2	116.2 (4)
C1—C2—C3	122.9 (6)	O8—C31—C32	122.0 (7)
C1—C2—Br1	121.2 (5)	O8—C31—C30	122.3 (7)
C3—C2—Br1	115.9 (4)	C32—C31—C30	115.6 (6)
O1—C3—C4	122.2 (6)	C33—C32—C31	123.0 (6)
O1—C3—C2	122.5 (6)	C33—C32—H32	118.5
C4—C3—C2	115.2 (5)	C31—C32—H32	118.5
C5—C4—C3	123.4 (6)	C32—C33—C34	121.8 (5)
C5—C4—H4	118.3	C32—C33—C44	122.4 (5)
C3—C4—H4	118.3	C34—C33—C44	115.7 (5)
C4—C5—C11	121.5 (5)	C33—C34—C29	113.1 (5)
C4—C5—C6	122.4 (5)	C33—C34—C35	105.8 (4)
C11—C5—C6	116.0 (5)	C29—C34—C35	105.6 (4)
C1—C6—C5	112.9 (5)	C33—C34—C36	108.5 (4)
C1—C6—C7	106.6 (4)	C29—C34—C36	109.6 (4)
C5—C6—C7	106.4 (5)	C35—C34—C36	114.3 (5)
C1—C6—C8	109.3 (4)	C34—C35—H35A	109.5
C5—C6—C8	108.4 (4)	C34—C35—H35B	109.5
C7—C6—C8	113.3 (4)	H35A—C35—H35B	109.5
C6—C7—H7A	109.5	C34—C35—H35C	109.5
C6—C7—H7B	109.5	H35A—C35—H35C	109.5
H7A—C7—H7B	109.5	H35B—C35—H35C	109.5
C6—C7—H7C	109.5	C37—C36—C42	114.8 (4)
H7A—C7—H7C	109.5	C37—C36—C34	115.3 (4)
H7B—C7—H7C	109.5	C42—C36—C34	110.0 (4)
C9—C8—C12	114.4 (4)	C37—C36—Cl2	103.0 (4)
C9—C8—C6	111.0 (4)	C42—C36—Cl2	107.3 (3)
C12—C8—C6	114.4 (4)	C34—C36—Cl2	105.5 (4)
C9—C8—Cl1	108.4 (3)	O9—C37—C38	112.5 (4)
C12—C8—Cl1	103.4 (3)	O9—C37—C36	106.3 (4)
C6—C8—Cl1	104.3 (3)	C38—C37—C36	114.0 (4)
C16—C9—C8	110.8 (4)	O9—C37—H37	107.9
C16—C9—C10	111.1 (4)	C38—C37—H37	107.9
C8—C9—C10	111.9 (4)	C36—C37—H37	107.9
C16—C9—H9a	107.6	C39—C38—C37	113.5 (4)
C8—C9—H9a	107.6	C39—C38—H38A	108.9
C10—C9—H9a	107.6	C37—C38—H38A	108.9
C11—C10—C9	112.3 (4)	C39—C38—H38B	108.9
C11—C10—H10A	109.1	C37—C38—H38B	108.9
C9—C10—H10A	109.1	H38A—C38—H38B	107.7
C11—C10—H10B	109.1	C38—C39—C40	111.5 (4)
C9—C10—H10B	109.1	C38—C39—C41	108.2 (4)
H10A—C10—H10B	107.9	C40—C39—C41	112.8 (4)
C5—C11—C10	111.1 (5)	C38—C39—C48	116.2 (4)
C5—C11—H11A	109.4	C40—C39—C48	108.2 (4)
C10—C11—H11A	109.4	C41—C39—C48	99.4 (4)
C5—C11—H11B	109.4	C39—C40—H40A	109.5
C10—C11—H11B	109.4	C39—C40—H40B	109.5
H11A—C11—H11B	108.0	H40A—C40—H40B	109.5
O2—C12—C8	108.2 (4)	C39—C40—H40C	109.5
O2—C12—C13	111.1 (4)	H40A—C40—H40C	109.5
C8—C12—C13	112.4 (4)	H40B—C40—H40C	109.5
O2—C12—H12	108.3	C45—C41—C42	118.0 (4)
C8—C12—H12	108.3	C45—C41—C39	103.4 (4)
C13—C12—H12	108.3	C42—C41—C39	112.6 (4)
C14—C13—C12	114.5 (4)	C45—C41—H41	107.4
C14—C13—H13A	108.6	C42—C41—H41	107.4
C12—C13—H13A	108.6	C39—C41—H41	107.4
C14—C13—H13B	108.6	C43—C42—C41	111.5 (4)
C12—C13—H13B	108.6	C43—C42—C36	111.9 (4)
H13A—C13—H13B	107.6	C41—C42—C36	110.6 (4)
C13—C14—C15	111.6 (4)	C43—C42—H42	107.5
C13—C14—C16	108.1 (4)	C41—C42—H42	107.5
C15—C14—C16	113.2 (4)	C36—C42—H42	107.5
C13—C14—C19	116.2 (4)	C44—C43—C42	113.1 (4)
C15—C14—C19	107.8 (4)	C44—C43—H43A	109.0
C16—C14—C19	99.6 (4)	C42—C43—H43A	109.0
C14—C15—H15A	109.5	C44—C43—H43B	109.0
C14—C15—H15B	109.5	C42—C43—H43B	109.0
H15A—C15—H15B	109.5	H43A—C43—H43B	107.8
C14—C15—H15C	109.5	C33—C44—C43	112.4 (5)
H15A—C15—H15C	109.5	C33—C44—H44A	109.1
H15B—C15—H15C	109.5	C43—C44—H44A	109.1
C17—C16—C9	120.2 (4)	C33—C44—H44B	109.1
C17—C16—C14	102.4 (4)	C43—C44—H44B	109.1
C9—C16—C14	113.6 (4)	H44A—C44—H44B	107.8
C17—C16—H16	106.6	C41—C45—C46	103.4 (4)
C9—C16—H16	106.6	C41—C45—H45A	111.1
C14—C16—H16	106.6	C46—C45—H45A	111.1
C16—C17—C18	104.2 (4)	C41—C45—H45B	111.1
C16—C17—H17A	110.9	C46—C45—H45B	111.1
C18—C17—H17A	110.9	H45A—C45—H45B	109.0
C16—C17—H17B	110.9	C47—C46—C45	111.8 (4)
C18—C17—H17B	110.9	C47—C46—C48	118.9 (5)
H17A—C17—H17B	108.9	C45—C46—C48	106.5 (4)
C20—C18—C17	114.5 (4)	C47—C46—H46	106.3
C20—C18—C19	117.5 (4)	C45—C46—H46	106.3
C17—C18—C19	105.6 (4)	C48—C46—H46	106.3
C20—C18—H18	106.1	C46—C47—H47A	109.5
C17—C18—H18	106.1	C46—C47—H47B	109.5
C19—C18—H18	106.1	H47A—C47—H47B	109.5
O6—C19—C21	109.3 (4)	C46—C47—H47C	109.5
O6—C19—C14	103.8 (4)	H47A—C47—H47C	109.5
C21—C19—C14	111.9 (4)	H47B—C47—H47C	109.5
O6—C19—C18	107.2 (4)	O13—C48—C49	107.6 (4)
C21—C19—C18	119.3 (4)	O13—C48—C46	108.3 (4)
C14—C19—C18	104.2 (4)	C49—C48—C46	119.5 (4)
C18—C20—H20A	109.5	O13—C48—C39	105.4 (4)
C18—C20—H20B	109.5	C49—C48—C39	111.6 (4)
H20A—C20—H20B	109.5	C46—C48—C39	103.7 (4)
C18—C20—H20C	109.5	O10—C49—C48	122.1 (5)
H20A—C20—H20C	109.5	O10—C49—C50	120.1 (5)
H20B—C20—H20C	109.5	C48—C49—C50	117.6 (4)
O3—C21—C19	123.1 (5)	O11—C50—C49	110.9 (4)
O3—C21—C22	119.7 (5)	O11—C50—H50A	109.4
C19—C21—C22	116.9 (4)	C49—C50—H50A	109.4
O4—C22—C21	110.0 (4)	O11—C50—H50B	109.4
O4—C22—H22A	109.7	C49—C50—H50B	109.4
C21—C22—H22A	109.7	H50A—C50—H50B	108.0
O4—C22—H22B	109.7	O12—C51—O11	123.2 (5)
C21—C22—H22B	109.7	O12—C51—C52	126.6 (5)
H22A—C22—H22B	108.2	O11—C51—C52	110.2 (5)
O5—C23—O4	121.6 (5)	C51—C52—C53	112.7 (5)
O5—C23—C24	127.5 (5)	C51—C52—H52A	109.1
O4—C23—C24	110.9 (5)	C53—C52—H52A	109.1
C23—C24—C25	113.7 (5)	C51—C52—H52B	109.1
C23—C24—H24A	108.8	C53—C52—H52B	109.1
C25—C24—H24A	108.8	H52A—C52—H52B	107.8
C23—C24—H24B	108.8	C52—C53—H53A	109.5
C25—C24—H24B	108.8	C52—C53—H53B	109.5
H24A—C24—H24B	107.7	H53A—C53—H53B	109.5
C24—C25—H25A	109.5	C52—C53—H53C	109.5
C24—C25—H25B	109.5	H53A—C53—H53C	109.5
H25A—C25—H25B	109.5	H53B—C53—H53C	109.5
C24—C25—H25C	109.5	O14—C54—O13	121.9 (5)
H25A—C25—H25C	109.5	O14—C54—C55	125.8 (5)
H25B—C25—H25C	109.5	O13—C54—C55	112.2 (4)
O7—C26—O6	122.4 (5)	C54—C55—C56	111.7 (4)
O7—C26—C27	126.1 (5)	C54—C55—H55A	109.3
O6—C26—C27	111.5 (5)	C56—C55—H55A	109.3
C26—C27—C28	114.4 (4)	C54—C55—H55B	109.3
C26—C27—H27A	108.6	C56—C55—H55B	109.3
C28—C27—H27A	108.6	H55A—C55—H55B	107.9
C26—C27—H27B	108.6	C55—C56—H56A	109.5
C28—C27—H27B	108.6	C55—C56—H56B	109.5
H27A—C27—H27B	107.6	H56A—C56—H56B	109.5
C27—C28—H28A	109.5	C55—C56—H56C	109.5
C27—C28—H28B	109.5	H56A—C56—H56C	109.5
H28A—C28—H28B	109.5	H56B—C56—H56C	109.5
			
C6—C1—C2—C3	−1.3 (9)	C34—C29—C30—C31	−1.7 (9)
C6—C1—C2—Br1	178.3 (4)	C34—C29—C30—Br2	177.6 (4)
C1—C2—C3—O1	175.1 (6)	C29—C30—C31—O8	−179.9 (6)
Br1—C2—C3—O1	−4.5 (9)	Br2—C30—C31—O8	0.8 (8)
C1—C2—C3—C4	−3.0 (9)	C29—C30—C31—C32	−2.9 (9)
Br1—C2—C3—C4	177.3 (4)	Br2—C30—C31—C32	177.8 (5)
O1—C3—C4—C5	−175.9 (6)	O8—C31—C32—C33	179.7 (6)
C2—C3—C4—C5	2.2 (9)	C30—C31—C32—C33	2.7 (9)
C3—C4—C5—C11	−179.7 (6)	C31—C32—C33—C34	2.0 (9)
C3—C4—C5—C6	2.9 (10)	C31—C32—C33—C44	178.6 (6)
C2—C1—C6—C5	6.0 (7)	C32—C33—C34—C29	−6.2 (8)
C2—C1—C6—C7	−110.5 (6)	C44—C33—C34—C29	177.1 (5)
C2—C1—C6—C8	126.7 (5)	C32—C33—C34—C35	108.9 (6)
C4—C5—C6—C1	−6.8 (8)	C44—C33—C34—C35	−67.8 (5)
C11—C5—C6—C1	175.7 (5)	C32—C33—C34—C36	−128.0 (6)
C4—C5—C6—C7	109.8 (6)	C44—C33—C34—C36	55.3 (6)
C11—C5—C6—C7	−67.7 (6)	C30—C29—C34—C33	6.1 (8)
C4—C5—C6—C8	−128.0 (6)	C30—C29—C34—C35	−109.1 (6)
C11—C5—C6—C8	54.5 (6)	C30—C29—C34—C36	127.3 (6)
C1—C6—C8—C9	−177.2 (4)	C33—C34—C36—C37	170.9 (5)
C5—C6—C8—C9	−53.8 (6)	C29—C34—C36—C37	47.0 (6)
C7—C6—C8—C9	64.1 (5)	C35—C34—C36—C37	−71.3 (6)
C1—C6—C8—C12	51.5 (6)	C33—C34—C36—C42	−57.4 (6)
C5—C6—C8—C12	174.9 (4)	C29—C34—C36—C42	178.7 (4)
C7—C6—C8—C12	−67.3 (6)	C35—C34—C36—C42	60.4 (5)
C1—C6—C8—Cl1	−60.7 (4)	C33—C34—C36—Cl2	58.0 (5)
C5—C6—C8—Cl1	62.7 (5)	C29—C34—C36—Cl2	−65.9 (5)
C7—C6—C8—Cl1	−179.4 (4)	C35—C34—C36—Cl2	175.8 (3)
C12—C8—C9—C16	−48.9 (6)	C42—C36—C37—O9	−81.9 (5)
C6—C8—C9—C16	179.8 (4)	C34—C36—C37—O9	47.6 (6)
Cl1—C8—C9—C16	65.8 (5)	Cl2—C36—C37—O9	161.9 (3)
C12—C8—C9—C10	−173.5 (4)	C42—C36—C37—C38	42.7 (6)
C6—C8—C9—C10	55.2 (6)	C34—C36—C37—C38	172.1 (4)
Cl1—C8—C9—C10	−58.8 (5)	Cl2—C36—C37—C38	−73.6 (5)
C16—C9—C10—C11	−178.4 (5)	O9—C37—C38—C39	72.8 (5)
C8—C9—C10—C11	−54.0 (6)	C36—C37—C38—C39	−48.3 (6)
C4—C5—C11—C10	128.5 (6)	C37—C38—C39—C40	−68.4 (6)
C6—C5—C11—C10	−54.0 (7)	C37—C38—C39—C41	56.2 (5)
C9—C10—C11—C5	51.4 (7)	C37—C38—C39—C48	166.9 (4)
C9—C8—C12—O2	−77.7 (5)	C38—C39—C41—C45	170.5 (4)
C6—C8—C12—O2	52.0 (5)	C40—C39—C41—C45	−65.7 (5)
Cl1—C8—C12—O2	164.7 (3)	C48—C39—C41—C45	48.8 (5)
C9—C8—C12—C13	45.3 (6)	C38—C39—C41—C42	−60.9 (5)
C6—C8—C12—C13	175.0 (4)	C40—C39—C41—C42	62.9 (5)
Cl1—C8—C12—C13	−72.2 (4)	C48—C39—C41—C42	177.3 (4)
O2—C12—C13—C14	72.4 (5)	C45—C41—C42—C43	−58.4 (6)
C8—C12—C13—C14	−49.0 (6)	C39—C41—C42—C43	−178.8 (4)
C12—C13—C14—C15	−70.9 (5)	C45—C41—C42—C36	176.4 (4)
C12—C13—C14—C16	54.1 (5)	C39—C41—C42—C36	56.0 (6)
C12—C13—C14—C19	164.9 (4)	C37—C36—C42—C43	−170.9 (5)
C8—C9—C16—C17	177.9 (4)	C34—C36—C42—C43	57.1 (6)
C10—C9—C16—C17	−57.0 (6)	Cl2—C36—C42—C43	−57.1 (5)
C8—C9—C16—C14	56.3 (6)	C37—C36—C42—C41	−45.9 (6)
C10—C9—C16—C14	−178.7 (5)	C34—C36—C42—C41	−177.9 (4)
C13—C14—C16—C17	170.5 (4)	Cl2—C36—C42—C41	67.9 (4)
C15—C14—C16—C17	−65.4 (5)	C41—C42—C43—C44	−176.1 (5)
C19—C14—C16—C17	48.8 (5)	C36—C42—C43—C44	−51.6 (7)
C13—C14—C16—C9	−58.4 (6)	C32—C33—C44—C43	133.0 (6)
C15—C14—C16—C9	65.7 (6)	C34—C33—C44—C43	−50.3 (7)
C19—C14—C16—C9	179.9 (4)	C42—C43—C44—C33	46.7 (7)
C9—C16—C17—C18	−170.0 (4)	C42—C41—C45—C46	−166.4 (4)
C14—C16—C17—C18	−42.9 (5)	C39—C41—C45—C46	−41.3 (5)
C16—C17—C18—C20	150.3 (4)	C41—C45—C46—C47	148.0 (4)
C16—C17—C18—C19	19.3 (5)	C41—C45—C46—C48	16.7 (5)
C26—O6—C19—C21	67.2 (5)	C54—O13—C48—C49	66.3 (5)
C26—O6—C19—C14	−173.3 (4)	C54—O13—C48—C46	−64.1 (5)
C26—O6—C19—C18	−63.4 (5)	C54—O13—C48—C39	−174.5 (4)
C13—C14—C19—O6	−39.6 (5)	C47—C46—C48—O13	134.3 (5)
C15—C14—C19—O6	−165.7 (4)	C45—C46—C48—O13	−98.5 (4)
C16—C14—C19—O6	76.1 (4)	C47—C46—C48—C49	10.8 (7)
C13—C14—C19—C21	78.1 (5)	C45—C46—C48—C49	138.0 (5)
C15—C14—C19—C21	−48.0 (5)	C47—C46—C48—C39	−114.1 (5)
C16—C14—C19—C21	−166.3 (4)	C45—C46—C48—C39	13.1 (5)
C13—C14—C19—C18	−151.7 (4)	C38—C39—C48—O13	−39.2 (5)
C15—C14—C19—C18	82.2 (4)	C40—C39—C48—O13	−165.5 (4)
C16—C14—C19—C18	−36.0 (5)	C41—C39—C48—O13	76.6 (4)
C20—C18—C19—O6	132.2 (4)	C38—C39—C48—C49	77.3 (5)
C17—C18—C19—O6	−98.6 (4)	C40—C39—C48—C49	−49.0 (5)
C20—C18—C19—C21	7.4 (6)	C41—C39—C48—C49	−167.0 (4)
C17—C18—C19—C21	136.7 (5)	C38—C39—C48—C46	−152.9 (4)
C20—C18—C19—C14	−118.2 (4)	C40—C39—C48—C46	80.8 (5)
C17—C18—C19—C14	11.0 (5)	C41—C39—C48—C46	−37.1 (5)
O6—C19—C21—O3	−161.9 (5)	O13—C48—C49—O10	−159.1 (4)
C14—C19—C21—O3	83.8 (6)	C46—C48—C49—O10	−35.2 (7)
C18—C19—C21—O3	−38.1 (7)	C39—C48—C49—O10	85.8 (6)
O6—C19—C21—C22	24.2 (5)	O13—C48—C49—C50	24.9 (6)
C14—C19—C21—C22	−90.2 (5)	C46—C48—C49—C50	148.8 (4)
C18—C19—C21—C22	147.9 (4)	C39—C48—C49—C50	−90.2 (5)
C23—O4—C22—C21	−76.7 (5)	C51—O11—C50—C49	−78.9 (5)
O3—C21—C22—O4	−7.2 (7)	O10—C49—C50—O11	−4.3 (7)
C19—C21—C22—O4	167.0 (4)	C48—C49—C50—O11	171.7 (4)
C22—O4—C23—O5	−7.2 (7)	C50—O11—C51—O12	−4.2 (7)
C22—O4—C23—C24	172.7 (4)	C50—O11—C51—C52	175.8 (4)
O5—C23—C24—C25	−16.9 (8)	O12—C51—C52—C53	−18.3 (8)
O4—C23—C24—C25	163.2 (5)	O11—C51—C52—C53	161.7 (5)
C19—O6—C26—O7	−10.5 (7)	C48—O13—C54—O14	−10.4 (7)
C19—O6—C26—C27	167.5 (4)	C48—O13—C54—C55	172.9 (4)
O7—C26—C27—C28	−149.2 (5)	O14—C54—C55—C56	20.5 (7)
O6—C26—C27—C28	32.9 (7)	O13—C54—C55—C56	−162.9 (4)

### Hydrogen-bond geometry (Å, °)

**Table:**

*D*—H···*A*	*D*—H	H···*A*	*D*···*A*	*D*—H···*A*
O2—H2···O7^i^	0.82	2.11	2.817 (5)	145
O9—H9···O14^ii^	0.82	1.98	2.754 (5)	158

Symmetry codes: (i) −*x*, *y*+1/2, −*z*; (ii) −*x*+2, *y*−1/2, −*z*+1.
